# A case report of immune-mediated arthritis in a patient with cutaneous melanoma receiving checkpoint inhibition therapy

**DOI:** 10.1097/MD.0000000000019439

**Published:** 2020-03-06

**Authors:** George Papaxoinis, Amalia Anastasopoulou, Katerina Laskari, Panagiotis Diamantopoulos, Olga Benopoulou, Helen Gogas

**Affiliations:** a First Department of Medicine, Laiko General Hospital, School of Medicine, National and Kapodistrian University of Athens; b Rheumatology Unit, First Department of Propaedeutic Internal Medicine, Joint Academic Rheumatology Program, Medical School, University of Athens, Athens, Greece.

**Keywords:** corticosteroids, hydroxychloroquine, immune checkpoint inhibitors, immune-mediated arthritis, leflunomide

## Abstract

**Introduction:**

Immune checkpoint inhibitors (ICIs) represent an important advance in the treatment of melanoma. ICIs may induce autoimmune phenomena caused by concurrent activation of the immune system against normal cells. During the last years, cases of musculoskeletal side effects, especially immune-mediated arthritis (IA), have been increasingly reported.

**Patient concerns:**

We present a 59-year-old woman, who was treated with pembrolizumab for a relapsed *BRAF* V600E mutated cutaneous malignant melanoma. The patient presented with right knee arthritis on week 30.

**Diagnosis:**

The erythrocyte sedimentation rate and serum C-reactive protein levels were elevated, while rheumatoid factor and anti-cyclic citrullinated peptide antibodies were negative. Imaging confirmed the presence of fluid mainly in the suprapatellar bursa. Synovial fluid analysis revealed an inflammatory effusion, while other etiologies of inflammatory arthritis were excluded.

**Interventions:**

Arthritis improved with an intra-articular injection of 8 mg dexamethasone. Twelve days later the arthritis relapsed in both knees, and although it was resistant to nonsteroidal anti-inflammatory treatment, it improved with systemic steroids. Tapering of methylprednisolone dose was feasible with the coadministration of leflunomide and subsequently hydroxychloroquine.

**Outcomes:**

Arthritis resolved and the patient is free of complications and disease activity 20 months after the initiation of the second line systemic treatment.

**Conclusions:**

We present an unusual case of IA associated with pembrolizumab treatment. The originality of the current report is based on the late occurrence, the monoarticular initial distribution, and uncommon location of IA at the knee.

## 1 Introduction

The introduction of immune checkpoint inhibitors (ICIs) in the treatment of advanced melanoma represents one of the most striking advances of recent decades.^[1]^ ICIs are monoclonal antibodies that target the immune checkpoint molecules that exist on the surface of immune cells and regulate their activity.^[1]^ Currently approved ICIs for melanoma are ipilimumab that targets cytotoxic T-lymphocyte-associated protein 4 (CTLA-4) and nivolumab and pembrolizumab that target programmed cell death protein 1 (PD-1). By inhibiting these checkpoint molecules ICIs aim at activating the immune system against tumor cells.^[1]^ Since the publication of the first clinical trial showing unprecedented efficacy in metastatic melanoma,^[2]^ many thousands of melanoma patients around the world have been treated with these agents. The most important side effects of ICIs are autoimmune phenomena caused by concurrent activation of the immune system against normal cells.^[3]^ Theoretically, any organ may be affected. Most commonly affected is the skin, the gastrointestinal, endocrine, and respiratory systems, and less commonly the nervous and cardiovascular systems.^[3]^

Although the musculoskeletal system is commonly affected in autoimmune diseases, no side effects related to ICIs are usually reported in the joints and muscles.^[3]^ An explanation for this may be the underestimation of these adverse events (AEs) in clinical trials, because they are usually mild. However, during the last couple of years cases of musculoskeletal side effects, especially immune-mediated arthritis (IA), have been increasingly reported.

In the current report, we describe the case of a female patient with advanced melanoma who developed IA after ICI treatment. Consent for this publication was obtained from the patient.

## 2 Case description

We present a 59-year-old woman of Belgian ancestry with negative past medical history, who was diagnosed with cutaneous malignant melanoma in the sole of the left foot. At the time of diagnosis, clinical examination and computed tomography scan of the chest, abdomen, and pelvis did not reveal findings suggestive of metastatic disease. The primary lesion was resected and histology demonstrated a nevoid melanoma of Breslow thickness 3.4 mm with ulceration. A second operation was then performed to have a wider margin width, as well as to resect a satellite lesion that had been found to be infiltrated by melanoma cells, and to examine sentinel lymph nodes, which were free of tumor. The pathology stage by AJCC (7th edition) was pT3bN2cM0 (stage IIIb).

The patient agreed to be enrolled in a randomized placebo-controlled phase III trial (CA209238) comparing Ipilimumab 10 mg/kg every 3 weeks for 4 doses, then every 12 weeks, to Nivolumab 3 mg/kg every 2 weeks as adjuvant treatment in high risk completely resected melanoma. Two months after enrollment, the patient developed grade 3 diarrhea due to autoimmune colitis. The treatment was stopped and she was started on oral methylprednisolone 1 mg/kg daily. Due to the severity of diarrhea, the patient was placed off protocol, and subsequent unblinding showed that she had been allocated to the Ipilimumab arm.

Nine months after discontinuing treatment the patient developed melanoma distant relapse with 2 subcutaneous lesions. Molecular tumor analysis showed a *BRAF* V600E mutation. Treatment with pembrolizumab 200 mg every 3 weeks was initiated. Clinical and imaging examinations on weeks 12 and 24 demonstrated partial tumor response, whereas on weeks 21 and 24 her treatment was deferred due to pancreatitis grade 2 that was well controlled with prednisolone 7.5 mg daily.

On week 30, the patient presented pain and edema of the right knee that occurred the day after the last pembrolizumab infusion. Clinical examination confirmed knee arthritis with swelling, increased temperature, and accumulation of intra-articular fluid. The knee movements were restricted due to pain. The patient was in good general condition with normal body temperature. Blood examinations showed grade 1 anemia (Hemoglobin [Hb] = 11.2 g/dL), a slightly elevated serum lactate dehydrogenase (247 U/L, upper normal limit 220 U/L), elevated erythrocyte sedimentation rate (100 mm/h), and serum C-reactive protein levels (63 mg/L, upper normal limit = 6 mg/L), while the white blood cell and platelet counts were within normal limits. A computed tomography of the right knee confirmed the presence of fluid in the suprapatellar bursa. A magnetic resonance scan of the right knee confirmed the presence of fluid mainly in the suprapatellar bursa and additionally showed degenerative lesions of both menisci with bucket handle tear of the medial meniscus. A synovial fluid analysis revealed an inflammatory effusion (7.200 cells/mm^3^). The Gram stain was negative, no crystals were detected, and the cultures for common pathogens and mycobacteria were negative. Also, the peripheral blood and urine cultures were negative. Immunological serum tests revealed a borderline titer of antinuclear antibodies (1:80), serum C3 levels were normal (161 mg/dL, normal limits 90–180 mg/dL), serum C4 levels were slightly elevated (52.5 mg/dL, normal limits 10–40 mg/dL), and rheumatoid factor (RF) and anti-cyclic citrullinated peptide (CCP) antibodies were negative. An intra-articular injection of 8 mg dexamethasone was performed and the arthritis improved. The patient was discharged with the prescription of lornoxicam 8 mg po daily.

Twelve days after discharge the arthritis relapsed with deterioration of the pain and swelling in both knees. The clinical examination suggested the presence of intra-articular fluid in both knees. Following a rheumatological consultation, the patient received etoricoxib 90 mg daily without improvement and finally responded to methylprednisolone 16 mg po daily on week 36.

At that time, a restaging computed tomography scan confirmed that the tumor was still in partial remission. Because of the BRAF mutation, it was decided to proceed to a second line systemic treatment with dabrafenib and trametinib. In spite of this treatment, the tapering of the methylprednisolone dose resulted in the relapse of joint pain; therefore, leflunomide 20 mg po daily was initiated. A gradual methylprednisolone tapering was then feasible and leflunomide was replaced by hydroxychloroquine 400 mg po daily because of mild elevation of the liver enzymes. The arthritis resolved and the patient is free of complications and disease activity 20 months after the initiation of the second line systemic treatment.

## 3 Discussion

IA represents a relatively rare complication of ICIs treatment. Inamo et al^[4]^ reported 3 cases out of 133 patients on ICIs having articular manifestations (2.3%), Lidar et al^[5]^ 14 out of 400 patients (3.5%), and Kostine et al^[6]^ 9 out of 524 patients (1.7%). Notably, of 36,973 ICI-related AEs recorded in the World Health Organization (WHO) database, only 86 (0.23%) were IA.^[7]^ A preponderance of age or gender with regard to IA has not been demonstrated.^[7,8]^

Our patient developed IA on the 30th week of treatment, which is a late occurring complication compared to other reports. In the largest study by Arnaud et al^[7]^ a median time of 2.7 months until the onset of IA is reported, while others report a median time of 2.3 to 4.8 months.^[6,8–12]^ A few reports refer to a longer median time of 7.5 to 11.2 months from treatment initiation to the development of IA.^[5,13,14]^ Interestingly, in the study of Pundole et al^[15]^ the median time from ICIs initiation to IA was 3 months in patients who developed polyarthritis or oligoarthritis, while it was 9 months in cases of monoarthritis.

IA may present as polyarthritis, oligoarthritis, or monoarthritis. Our patient initially developed monoarthritis which evolved to oligoarthritis. With regard to the distribution of the affected joints, there is great heterogeneity reported in previous publications. In one of the largest studies,^[8]^ almost all patients had polyarticular involvement, mostly symmetric. In contrast, in the case series described by Leipe et al^[11]^ the majority of patients had monoarthritis. In a systematic review by Pundole et al^[15]^ only 8 out of 90 cases (8.9%) had monoarthritis. Notably, in 87.5% of these patients the shoulder was the involved joint.

We should emphasize that IA associated with ICI treatment is rarely related to a systemic autoimmune disease. The majority of patients have no medical history of an autoimmune disease. Moreover, de novo cases of seropositive (RF and/or anti-CCP antibodies) IA due to ICIs have been very rarely reported.^[11,14,16]^ Indeed, our patient was negative for autoantibodies and did not fulfill the 2010 Rheumatoid arthritis classification criteria.^[17]^ We should also notify that our patient developed, beside IA, other immune-mediated AEs, such as pancreatitis. However, no other signs or symptoms of a systemic autoimmune disease were evident. Interestingly, Lidar et al^[5]^ observed that 50% of patients with musculoskeletal AEs had immune-mediated AEs also in other organs.

With regard to treatment, IA is successfully treated with systemic corticosteroids.^[8,11,15]^ Also, the patients may benefit from an additional local therapy with intra-articular corticosteroid injections. However, IA relapses occur in the majority of cases after corticosteroid dose tapering.^[11]^ In resistant cases, methotrexate^[11]^ or hydroxychloroquine^[14]^ is most commonly used in order to wean from corticosteroids. Our patient deteriorated after local steroid treatment and lornoxicam, and showed a poor response to etoricoxib, while she responded to systemic steroids. Tapering was successful with the concurrent use of leflunomide and subsequently hydroxychloroquine. There are no definite guidelines on the continuation or not of ICIs in cases with drug-related complications.^[3,18]^ In our patient, the ICI treatment was discontinued because of the occurrence of 2 immune-mediated AEs.

With regard to other antineoplastic agents, IA arthritis has been more frequently observed as a side effect of anti-PD-1 ICI agents,^[15,19]^ and it may also develop after the administration of anti-CTLA-4 antibodies. However, the higher rate of anti-PD-1-related IA reported in literature may be due to their much more frequent use. Nevertheless, our patient developed IA while on pembrolizumab and not after ipilimumab treatment. Finally, whether treatment with kinase inhibitors, which our patient received at last, may improve articular symptoms, needs further evaluation. Nevertheless, in our hands, the arthritis did not ameliorate after the initiation of this treatment.

## 4 Conclusion

To summarize, we present a case of IA after treatment with pembrolizumab, which initially manifested as knee monoarthritis. The arthritis evolved to oligoarthritis in spite of local corticosteroid and nonsteroidal anti-inflammatory treatment. The patient finally responded to systemic steroids but dose tapering was feasible without symptom recurrence with the concurrent use of leflunomide/hydroxychloroquine. In conclusion, in such cases, the close cooperation between oncologists and rheumatologists is essential.

## Author contributions

**Conceptualization:** George Papaxoinis, Amalia Anastasopoulou, Helen Gogas.

**Data curation:** George Papaxoinis, Amalia Anastasopoulou, Katerina Laskari, Panagiotis Diamantopoulos, Olga Benopoulou, Helen Gogas.

**Formal analysis:** George Papaxoinis, Amalia Anastasopoulou, Katerina Laskari.

**Investigation:** George Papaxoinis, Amalia Anastasopoulou, Katerina Laskari, Panagiotis Diamantopoulos, Olga Benopoulou, Helen Gogas.

**Supervision:** Olga Benopoulou, Helen Gogas.

**Validation:** George Papaxoinis, Amalia Anastasopoulou, Katerina Laskari, Panagiotis Diamantopoulos, Olga Benopoulou, Helen Gogas.

**Writing – original draft:** George Papaxoinis, Amalia Anastasopoulou, Katerina Laskari.

**Writing – review & editing:** George Papaxoinis, Amalia Anastasopoulou, Katerina Laskari, Panagiotis Diamantopoulos, Olga Benopoulou, Helen Gogas.

George Papaxoinis orcid: 0000-0001-6900-784X.
